# Multi-year optimization of malaria intervention: a mathematical model

**DOI:** 10.1186/s12936-016-1182-0

**Published:** 2016-03-01

**Authors:** Harry J. Dudley, Abhishek Goenka, Cesar J. Orellana, Susan E. Martonosi

**Affiliations:** University of Colorado Boulder, 526 UCB, University of Colorado, Boulder, CO 80309-0526 USA; Harvey Mudd College, 301 Platt Blvd, Claremont, CA 91711 USA

**Keywords:** Malaria policy, Operations research, Compartment model, Integer programming

## Abstract

**Background:**

Malaria is a mosquito-borne, lethal disease that affects millions and kills hundreds of thousands of people each year, mostly children. There is an increasing need for models of malaria control. In this paper, a model is developed for allocating malaria interventions across geographic regions and time, subject to budget constraints, with the aim of minimizing the number of person-days of malaria infection.

**Methods:**

The model considers a range of several conditions: climatic characteristics, treatment efficacy, distribution costs, and treatment coverage. An expanded susceptible-infected-recovered compartment model for the disease dynamics is coupled with an integer linear programming model for selecting the disease interventions. The model produces an intervention plan for all regions, identifying which combination of interventions, with which level of coverage, to use in each region and year in a 5-year planning horizon.

**Results:**

Simulations using the model yield high-level, qualitative insights on optimal intervention policies: The optimal intervention policy is different when considering a 5-year time horizon than when considering only a single year, due to the effects that interventions have on the disease transmission dynamics. The vaccine intervention is rarely selected, except if its assumed cost is significantly lower than that predicted in the literature. Increasing the available budget causes the number of person-days of malaria infection to decrease linearly up to a point, after which the benefit of increased budget starts to taper. The optimal policy is highly dependent on assumptions about mosquito density, selecting different interventions for wet climates with high density than for dry climates with low density, and the interventions are found to be less effective at controlling malaria in the wet climates when attainable intervention coverage is 60 % or lower. However, when intervention coverage of 80 % is attainable, then malaria prevalence drops quickly in all geographic regions, even when factoring in the greater expense of the higher coverage against a constant budget.

**Conclusions:**

The model provides a qualitative decision-making tool to weigh alternatives and guide malaria eradication efforts. A one-size-fits-all campaign is found not to be cost-effective; it is better to consider geographic variations and changes in malaria transmission over time when determining intervention strategies.

## Background

Malaria remains a lethal disease affecting an estimated 200 million people and killing 627,000 in 2012 [1]. There are a variety of interventions for treating or preventing malaria infection, but the use of these interventions is hindered by scarcity of resources. Mathematical models provide a useful tool for evaluating intervention strategies and studying the relative effectiveness of interventions. These evaluations will become increasingly useful as success with malaria elimination is predicted to change transmission dynamics. In fact, the WHO Global Malaria Programme cites the specific need for operations research models to determine the best intervention strategies in areas where transmission dynamics are changing as malaria is being eliminated [2].

In this paper, an integer linear program (ILP) and a coupled susceptible-infected-recovered (SIR) compartment model are developed to create a decision-making tool for planning future interventions. The model suggests the best strategy for minimizing person-days of malaria infection over a 5-year period given an initial population, cost of each intervention, and a budget constraint. The model allows for the possibility of a malaria vaccine in combination with other interventions. Simulations are performed in which the budget, the efficacy of the interventions, and their cost are varied to determine the sensitivity of the optimal policy to these parameters.

### Interventions

There are many existing methods to prevent or treat malaria infection. The model will consider the following five interventions and their combinations.

*Long-lasting insecticidal nets* (LLINs) cover sleeping individuals during the night when mosquito biting can be highest. When intact, the nets block mosquitoes from reaching humans. The insecticides work by deterring mosquitoes from feeding and by killing female mosquitoes that come in contact with the net. LLINs can remain effective for multiple years [3]. In fact, the WHO Pesticide Evaluation Scheme 2005 guidelines state that LLINs should survive at least 3 years of recommended washing and use [4].

*Indoor residual spraying* (IRS) is another insecticidal prevention method. IRS is believed to deter mosquitoes from entering sprayed areas and to kill female *Anopheles* mosquitoes that rest on sprayed surfaces after feeding. (Resting after feeding is a hallmark of some mosquito species while others prefer to rest outdoors [5]). Historically, IRS with an insecticide called dichlorodiphenyltrichloroethane was effective in reducing malaria in Europe, Asia, and Latin America. However, as insecticide use increases, insecticide resistance has been observed in some mosquito populations in Africa, and new insecticides must be used [1].

*Intermittent preventive therapy* (IPT) is the regular administration of a drug like sulfadoxine–pyrimethamnine to decrease morbidity due to malaria in infants, children, and pregnant women. IPT decreases the chance of developing symptoms after being bitten by an infected mosquito [6]. There is evidence that children withstand acute infection better than adults. However, in endemic areas, adults develop acquired immunity from repeated exposures, and children remain more susceptible to high levels of parasitaemia (parasite density in the blood) [7]. Most of the 627,000 people killed by malaria in 2012 were children in Africa, so giving IPT to infants, children, and pregnant women treats the most vulnerable population while limiting the risk of spreading drug resistance [1].

*Artemisinin combination therapy* (ACT) can be used to treat a patient after they contract malaria. This is the best treatment for uncomplicated *P. falciparum* malaria when confirmed by rapid diagnostic tests (RDT) [1, 8]. ACT kills the parasites that cause symptoms and may destroy or disable the gametocytes that are responsible for infecting mosquitoes [9]. Both these factors mean that ACT increases the recovery rate.

Many *malaria vaccines* are in development, and one has gone through Phase III clinical trials. The complex life-cycle of the malaria parasite makes it possible to intervene at many stages. Vaccines that target different forms of the parasite will operate by different mechanisms, but in general, a vaccine would decrease the chance of developing symptoms and increase the recovery rate if infected. The leading malaria vaccine candidate is the RTS,S malaria vaccine. It is an antigen composed of the RTS and S proteins. The RTS,S vaccine is a pre-erythrocytic vaccine that presents circumsporozoite protein (CSP) from malaria sporozoites to the immune system. CSP is a parasitic surface protein that is an important part of the invasion of hepatocytes by sporozoites [10]. Such a vaccine will decrease the probability that a susceptible person becomes infected after a bite from an infectious mosquito. Moreover, it is believed the vaccine could increase a person’s recovery rate by increasing their exposure to asexual blood-stage parasites, thereby boosting their immunity [10]. (By contrast, a *transmission-blocking* vaccine that acts in mosquitoes would decrease the probability of transmission from an infectious mosquito but would not change the human recovery rate).

### Literature review

This paper extends a single-stage optimization model of Dimitrov et al. Their model divides the country of Nigeria into approximately 270,000 cells and chooses one action (either a single intervention or no intervention) for each cell over a year, subject to budget constraints, to minimize societal costs caused by malaria infection. The model also identifies optimal locations for supply distribution centres. They treat the societal benefit of each intervention as an exogenous parameter that depends on geographic characteristics. This allows their model to consider geographic variability in malaria dynamics [11].

However, because malaria dynamics depend on the fraction of the population that is infectious, a quantity that the interventions are themselves trying to reduce, the framework of Dimitrov *et al.* does not permit the examination of multiyear efforts against malaria in which the optimal policy might vary over time as the malaria dynamics shift. This paper extends the optimization model above to select interventions (or combinations thereof) over multiple years by explicitly incorporating malaria disease dynamics over time in response to those interventions. This is a novel approach that combines two areas of mathematics that do not regularly interact: ILP from the area of operations research and differential equations modelling from the area of mathematical epidemiology.

There is a long history of mathematical models of malaria transmission, going back to the work of Sir Ronald Ross in the early 1900s [12, 13]. In recent years, malaria has drawn significant attention from the academic community. Epidemiologists have traditionally modelled the spread of malaria in a population using variations on the SIR model to capture different aspects of the disease. Mandal et al. survey the models found in the literature and offer a hierarchy based on model complexity [13].

In order for the model presented here to make informed choices about which interventions to distribute, the dynamics of how disease transmission change after treatment interventions must first be understood. Lindblade et al. and Killeen et al. study the protective effect of insecticide-treated nets or LLINs [16]. Bousema et al. investigate how ACT reduces the circulation time of gametocytes, thereby reducing infectiousness [16]. Garner and Graves examine the community benefits of ACT [17]. Chandramohan et al., Grobusch et al., and Aponte *et al.* quantify the protective effects of IPT for infants [6, 18, 19]. Pluess et al. review the effects of IRS [5]. These results are used to inform the model’s choice of disease transmission parameters, as described later under “Effects of interventions on SIR parameters” section.

The model presented here includes in its portfolio of interventions a vaccine that is currently in development. Prosper et al. model the interaction between vaccine- and naturally-acquired immunity using a five-compartment model. Their model augments the *S*, *I*, and *R* classes with a partially-immune (due either to vaccination or natural immunity) susceptible class and a moderately-infectious class for infected, partially-immune individuals. They find that disease burden can be decreased only if a highly effective vaccine is coupled with a policy of actively treating asymptomatic infections in partially immune individuals [20]. Bojang et al. report there is minimal potential effect for a malaria vaccine given to adult men, and Asante et al. study the positive potential protective benefits of administering the vaccine to children [21, 22].

There are extensions to the SIR framework that are not considered here. Koella and Antia model the reduced efficacy of interventions due to the spread of drug-resistant strains of malaria [23]. The model presented here does not incorporate drug-resistance, so any policy recommended by the model should be evaluated in this context. Other researchers, for example Dawes et al. [24] and Koudou et al. [25], focus on the mosquitoes’ plasmodial transmission dynamics by analysing the effects of interventions on mosquito morbidity and mortality rates and the usefulness of the resulting manipulation of said rates. The changing mosquito population is not modelled explicitly; instead the effects of interventions on the mosquito population are represented as changes in the parameter values used in the human SIR model.

While the above references provide detailed models of malaria’s complex dynamics, this paper presents a simple SIR model that accommodates the effects of several types of interventions, while maintaining the computational tractability required by the optimization model. In the next section, the model and simulation approach are described in greater detail.

## Methods

This paper considers the problem of allocating malaria treatments to many regions when limited by scarce resources. There is assumed to be a fixed annual budget shared across several geographic regions having different initial incidences and transmission rates of malaria and different unit costs for distributing treatment. A portfolio of interventions can be selected, including some in combination, each having its own effects on malaria transmission. Each intervention is selected at a particular *coverage*, which is the percentage of the population that receives the intervention and uses it correctly. Social and economic losses are assumed to be proportional to the time spent infectious, so person-days of malaria infection is the chosen measure of the malaria burden. The model identifies the optimal sequence of interventions and corresponding coverage percentages for each region and each year that minimizes the total infected person-days over a fixed time horizon.

An integer linear programming optimization model (ILP) suggests the best set of interventions in each year to minimize person-days of malaria infection over all time steps. The ILP takes as input the number of person-days of malaria infection that occur when a given intervention is used on a population with a given initial prevalence of malaria. The person-days of malaria infection is estimated by a SIR differential equations model of malaria transmission dynamics.

### Integer linear programming (ILP) model

The ILP relies on several sets, parameters, and decision variables, which are defined here.

#### Sets

*Geographic regions* Because the cost of distributing an intervention to a particular district depends on its infrastructure and ease of access to treatment, and the malaria transmission dynamics depend on its climate, districts are grouped into geographic regions, denoted by index *g*. The optimization model determines the number of districts in each geographic region to receive a particular sequence of interventions.

*Population states* A population state, *p*, is a triplet, (*S*, *I*, *R*), that indicates the percentage of a district’s population susceptible to (*S*), infected by (*I*), or recovered from and temporarily immune to (*R*) malaria. Each district begins a year in a particular population state and ends in a new population state that depends on how the chosen intervention affects the malaria disease dynamics. (The model for determining the disease progression is described in the “Differential equations (DE) model” section).

*Actions* The set of actions is the set of possible choices of intervention (including certain combinations of interventions, or the possibility of applying no intervention). The choice of intervention at a determined coverage level in a district is referred to as an action, denoted by index *i*.

#### Parameters

$$A^{in}_{igpq}$$
is an indicator variable whose value is 1 if action *i* applied to a district of geographic region *g*, initially in population state *p* causes a transition to population state *q*, and 0 otherwise.
$$A^{out}_{igp}$$
is an indicator variable whose value is 1 if action *i* applied to a district of geographic region *g*, initially in population state *p* causes a transition to a different population state, and 0 otherwise.
$$B_{t}$$
is the annual budget for year *t*; the combined cost of actions across all districts in year *t* must not exceed this value.
$$C_{ig}$$
is the cost of action *i* in any district in geographic region *g*.
$$I_{pg}$$
is the number of districts in geographic region *g* that are initially in population state *p* at the first time step.
$$L_{ipg}$$
is the number of person-days of malaria infection incurred in a district in geographic region *g*, initially in population state *p*, under action *i*.
*N*: is a number larger than the total number of population states.
*T*: is the time horizon, in years, considered by the model.

#### Decision variables

$$P_{pgt}$$
is the number of districts in geographic region *g* that are initially in population state *p* at the start of year *t*.
$$a^{OUT}_{ipgt}$$
is the number of districts in geographic region *g* that are initially in population state *p* at the start of year *t* and are assigned action *i*.
$$a^{IN}_{ipqgt}$$
is the number of districts in geographic region *g* that are initially in population state *p* at the start of year *t*, are assigned action *i*, and end in population state *q*.

#### Model

Using these sets, parameters and decision variables, the following ILP can now be defined.

1 $$\begin{aligned} min&\sum _{t,i,p,g} L_{ipg} a^{OUT}_{ipgt} \end{aligned}$$

2 $$\begin{aligned} s.t.&\sum _{i,g} C_{ig} \sum _{p} a^{OUT}_{ipgt} \le B_{t}&\forall t \end{aligned}$$

3 $$\begin{aligned}&P_{p,g,t=1} = I_{pg}&\forall p, g \end{aligned}$$

4 $$\begin{aligned}&\sum _{p}P_{pgt} = \sum _{p}P_{p,g,t+1}&\forall g, t \end{aligned}$$

5 $$\begin{aligned}&\sum _{i}a^{OUT}_{ipgt} = P_{pgt}&\forall p, g, t \end{aligned}$$

6 $$\begin{aligned}&P_{pgt} + \sum _{i,q} a^{IN}_{ipqgt} - \sum _{i} a^{OUT}_{ipgt} = P_{p,g,t+1}&\forall p, g, t \end{aligned}$$

7 $$\begin{aligned}&\sum _{p} a^{IN}_{ipqgt} = a^{OUT}_{iqgt}&\forall i, q, g, t \end{aligned}$$

8 $$\begin{aligned}&a^{IN}_{ipqgt} \le N \cdot A^{in}_{igpq}&\forall i, p, q, g, t \end{aligned}$$

9 $$\begin{aligned}&a^{OUT}_{ipgt} \le N \cdot A^{out}_{igp}&\forall i, p, g, t \end{aligned}$$

$$\begin{aligned}&P_{pgt}, a^{IN}_{ipqgt}, a^{OUT}_{ipgt} \ge 0, integer&\forall i, p, q, g, t \end{aligned}$$

The objective function in expression (1) minimizes the cumulative person-days that each district spends in the infected state over the time horizon, as a function of the model’s choice of actions. Constraint (2) requires the chosen set of interventions to be within budget in each year. Constraint (3) initializes the population variable at the start of the time horizon. Constraints (4), (5), (6) and (7) are bookkeeping constraints that keep track of the number of districts in each geographic region and population state as a function of the actions chosen. Constraints (8) and (9) assure that districts transition out of population state *p* to population state *q* only when an appropriate action has been taken. The last constraint requires all decision variables to be nonnegative integers.

### Differential equations (DE) model

Several of the parameters used by the ILP model, specifically $A^{in}_{igpq}$, $A^{out}_{igp}$ and $L_{ipg}$ depend on the dynamics of malaria progression. The SIR model is a standard system of nonlinear ordinary differential equations for analysing the transmission of malaria [13, 23]. In this paper, the standard model is modified to use a coupled six-class compartment model with separate SIR compartments for treated and untreated individuals. This coupling of treated and untreated SIR classes permits modelling of population-wide benefits caused by decreased infectiousness of a treated subpopulation. For an initial population state and action, this system of equations is solved to determine the population state after 1 year. This yields the indicator parameters $A^{in}_{igpq}$ and $A^{out}_{igp}$. The solution to this system of differential equations is also used to estimate the burden of malaria, measured in infected person-days, during that year. For each district in geographic region *g*, beginning the year in a particular population state *p*, having been assigned action *i*, the infected class curve that results under those conditions is numerically integrated, multiplied by the district’s population. This estimates the number of people who are infected over the year times the number of days for which they remain infected. This number is then input into the linear programming model as the value of $L_{ipg}$. This is pre-solved for all possible population states and actions, and the results are stored as input data for the ILP.

The parameters, state variables and system of differential equations are now defined.

#### Parameters

$$a_u$$ ($$a_t$$)
is the number of bites per mosquito per untreated (respectively, treated) human per day.
$$b_u$$ ($$b_t$$)
is the transmission efficacy from infected mosquito to susceptible, untreated (resp., treated) human.
*c*: is the transmission efficacy from infected human to susceptible mosquito.
$$\delta$$
is the daily birth rate and death rate. Constant population is assumed.
$$\gamma _u$$ ($$\gamma _t$$)
is the recovery rate for untreated (resp., treated) people. Its reciprocal is the average time that a person is infected with malaria.
$$h_u$$ ($$h_t$$)
is the *force of infection*, that is, the rate at which untreated (resp., treated) susceptible humans become infected with malaria.
$$m_u$$ ($$m_t$$)
is the number of mosquitoes per untreated (resp., treated) human.
$$\mu$$
is the mosquito mortality rate.
$$\omega$$
is the duration of immunity without reinfection.
*q*: is the treatment coverage, the percentage of the population that receives a treatment and uses it correctly. It is assumed that the same percentage of newborns are born into the susceptible, treated class. The remaining fraction, $1-q$, are born into the susceptible, untreated class.
$$\rho _u$$ ($$\rho _t$$)
is rate of immunity loss for recovered untreated (resp., treated) humans.
$$\tau$$
is the incubation period of malaria in the mosquito.

#### State variables

$$S_u$$ ($$S_t$$)
is the proportion of the population that is susceptible and untreated (resp., treated).
$$I_u$$ ($$I_t$$)
is the proportion of the population that is symptomatic, infectious, and untreated (resp., treated).
$$R_u$$ ($$R_t$$)
is the proportion of the population that is recovered with acquired immunity and untreated (resp., treated).

#### Model

The proportions of the population belonging to each of the six classes can be determined by solving the following system of differential equations:

10 $$\begin{aligned}&\frac{dS_u}{dt} = \delta (1-q) - (\delta + h_u)S_u + \rho _u R_u \end{aligned}$$

11 $$\begin{aligned}&\frac{dI_u}{dt} = h_u S_u - (\delta + \gamma _u )I_u \end{aligned}$$

12 $$\begin{aligned}&\frac{dR_u}{dt} = \gamma _uI_u - (\delta + \rho _u)R_u \end{aligned}$$

13 $$\begin{aligned}&\frac{dS_t}{dt} = \delta q - (\delta + h_t)S_t + \rho _t R_t \end{aligned}$$

14 $$\begin{aligned}&\frac{dI_t}{dt} = h_t S_t - (\delta + \gamma _t)I_t \end{aligned}$$

15 $$\begin{aligned}&\frac{dR_t}{dt} = \gamma _t I_t - (\delta + \rho _t)R_t. \end{aligned}$$

Although on the surface, the equations for the untreated population and the equations for the treated population do not appear to be coupled, the coupling occurs with the parameters $h_u$ and $h_t$, which are the force of infection parameters. They have been derived by Smith and McKenzie [26] to be:

16 $$\begin{aligned} h_u = \frac{m_u a_u^2 b_u c e^{-\mu \tau } (I_u + I_t)}{\mu + a_u c (I_u + I_t)} \end{aligned}$$

17 $$\begin{aligned} h_t = \frac{m_t a_t^2 b_t c e^{-\mu \tau } (I_u + I_t)}{\mu + a_t c (I_u + I_t)} . \end{aligned}$$

Observe that these rates are functions of the total proportion of infectious people, $I_u + I_t$, which couples the system of differential equations. The more infectious people there are in either the untreated or treated group, the faster the rate at which susceptible people in either group can become infected.

The rates of immunity loss, $\rho _u$ and $\rho _t$, are functions of $h_u$ and $h_t$, respectively and further couple the system. The procedure for deriving the rate of immunity loss has been shown by Aron and May [27]. These equations assume that being exposed to malaria while recovering resets the duration of immunity.

18 $$\begin{aligned} \rho _u = \frac{h_u+\delta }{e^{\omega (h_u+\delta )}-1} \end{aligned}$$

19 $$\begin{aligned} \rho _t = \frac{h_t+\delta }{e^{\omega (h_t+\delta )}-1} \end{aligned}$$

This is a general model that does not consider the effect an intervention can have on the transmission of the disease. In the specific case of ACT, a medication that clears infection rapidly, the length of time a malaria patient is carrying infectious gametocytes in her blood is significantly reduced [16, 28]. Because of this, the model makes the assumption that ACT clears parasites before the body has time to develop acquired immunity; therefore, infected people treated with ACT are assumed to skip the recovered class and transition directly back to the susceptible class. To reflect this, the indicator variable $\psi _{act}$ is introduced, which equals 1 when ACT is chosen (either alone or in combination with another intervention), and 0 otherwise. The state equations for the untreated class are unchanged, and the equations for the treated classes become:

20 $$\begin{aligned}&\frac{dS_t}{dt} = \delta q - (\delta + h_t)S_t + \rho _t R_t + \psi _{act} \gamma _t I_t \end{aligned}$$

21 $$\begin{aligned}&\frac{dI_t}{dt} = h_t S_t - (\delta + \gamma _t)I_t \end{aligned}$$

22 $$\begin{aligned}&\frac{dR_t}{dt} = (1-\psi _{act})\gamma _t I_t - (\delta + \rho _t)R_t \end{aligned}$$

Observe that when ACT is used, infectious individuals bypass the recovered class and transition directly to the susceptible class.

Because a new portfolio of interventions is selected each year, the effects of treatment are assumed to last for 1 year, exactly. Some of the treatments are known to last longer; for instance, the insecticide coating on mosquito nets is believed to be effective for 3 years, and vaccines in development currently have an efficacy of 3 years. However, assuming a duration of only 1 year is conservative: under this assumption, the model will underestimate the efficacy of the interventions, and the results expected to be seen in the field should be better. Under this assumption, at the end of each year, the six-state population $(S_u, I_u, R_u, S_t, I_t, R_t)$ can be collapsed into a more compact three-state representation: $(S_u+S_t, I_u+I_t, R_u+R_t).$

#### Coverage

The coverage, *q*, refers to the percentage of the population that receives a treatment and uses it correctly. For example, if at the start of the year, the percentages of the population who are susceptible, infected and recovered are given by (*S*, *I*, *R*), respectively, then the initial values of $S_u, I_u, R_u, S_t, I_t,$ and $R_t$ for the differential equations model will be $(1-q)S, (1-q)I, (1-q)R, qS, qI,$ and $qR$, respectively.

However, some interventions, such as IPT and vaccine, are assumed to be distributed only to newborns and children under the age of four. In these cases, the coverage, *q*, applies only to births and to the fraction of the population under the age of four. If *x* is the fraction of the population under the age of four, and (*S*, *I*, *R*) is the initial distribution of susceptible, infected and recovered individuals in the population, then the initial values of $S_u, I_u, R_u, S_t, I_t,$ and $R_t$ for the differential equations model will be $(1-qx)S, (1-qx)I, (1-qx)R, qxS, qxI,$ and *qxR*.

### Data

The model relies on parameters governing intervention costs, malaria transmission, and intervention efficacy. When available, parameter values are estimated based on malaria research literature. When using country-specific information, data from Kenya or its neighbours are used as it is more readily available and permits consistency across parameters. This paper also presents sensitivity analysis to understand how the model’s results would change under a range of scenarios concerning distribution costs, climate and intervention efficacy. In this section, the costs of the interventions are described first, followed by the baseline parameter values used in the SIR model. Then, the changes in these parameter values under interventions and sensitivity analysis scenarios are described.

#### Base costs of interventions

The model parameter $C_{ig}$ is the per person, per year cost of action *i* in any district in geographic region *g*. The cost of an action depends on the purchase price as well as transportation and distribution costs, which are assumed to be regional. For the base cost, the simulations use data provided by White et al., who survey cost and cost-effectiveness data for LLIN, IRS, IPT, and ACT from all available sources and adjust it to 2009 USD [29]. The simulations primarily use data from Kenya, except where noted that no Kenya-specific data was available; in these cases, cost estimates from nearby Ethiopia, Tanzania and Zimbabwe are used.

Listed here are the base costs for each intervention; the subsequent section describes how to modify those costs to reflect transportation and distribution costs in different geographic regions. These are summarized in Table 1.

**Table 1:** Baseline cost for using interventions, per treated person for 1 year, in 2009 USD [29–31]

Intervention	Cost
None	0
LLIN	1.33
ACT	4.82
IPT	1.13
IRS	2.22
Vaccine	20.66

*LLIN* The average cost of a single insecticide-treated mosquito net is 7.21 USD [29], and the WHO Pesticide Evaluation Scheme 2005 guidelines estimate a 3 year life span with recommended use [4]. Because the model assumes all actions expire at the end of 1 year, an annual cost per net of 2.40 USD is used, which is one-third the base cost of the net. Moreover, bed-sharing is a common practice that further reduces the per-person cost of each distributed net. The World Health Organization recommends the assumption that an LLIN will protect 1.8 people, on average [30], making the annual per-person cost 1.33 USD.

*IRS* The IRS cost estimate assumes two rounds of household spraying with lambda cyhalothrin per person per year, at an annual cost of 2.22 USD [29].

*IPT* White et al. summarize cost estimates for distributing IPT to newborns, children and pregnant women. The mean cost of distributing six bi-monthly doses of sulfadoxine–pyrimethamine to infants in Tanzania is reported to be 0.78 USD, and three doses per year to children in Kenya is 1.25 USD [29]. As roughly 25 % of children under the age of 4 are infants, the simulations use an estimated weighted average annual cost for IPT of 1.13 USD.

*ACT* White et al. report malaria diagnosis and treatment costs for a variety of diagnostic methods and treatment types in several countries. For consistency, the simulation uses costs associated specifically with RDT used in conjunction with ACT treatment in the countries of Tanzania and Zambia. These range from 3.63 USD to 6.72 USD, with an average of 4.82 USD per person treated [29]. Unlike interventions such as LLINs, which are assumed to be distributed to the entire treated class, ACT is distributed only to members of the treated class who experience a malaria infection. Therefore, the SIR model must estimate the number of new malaria infections per year to determine the annual cost of ACT. According to Eq. (21), new infections occur with rate $h_tS_t = \frac{dI_t}{dt} +(\delta +\gamma _t)I_t$. Note that $\frac{dI_t}{dt}$ can be approximated by $\frac{I_t(d+\epsilon )-I_t(d)}{\epsilon }$ for small $\epsilon$. Discretizing the year over which the treatment is available into 365 days and letting $\epsilon =1$ day, the number of new infections appearing on day *d* should be roughly $I_t(d) - \left( 1-(\delta +\gamma _t)\right) I_t(d-1)$ times the total population. Summing this value over all days *d* should give an approximation of the number of new infections incurred during the year, and hence, the number of people who received ACT.

*Vaccine* Cost data for the RTS,S vaccine is not yet available since the vaccine is not yet on the market. Seo et al. use an estimate of 7 USD per dose for the vaccine after looking at recent introductory vaccine prices ranging from 1 to 15 USD [31]. They also propose using 0.37 USD administration cost per vaccination based on the price for other vaccines used in Malawi in the Expanded Program on Immunization (EPI). Because the RTS,S vaccine is administered in three doses, they estimate the total cost of the vaccine at 22.11 USD per person per year [31]. Adjusting their 2012 costs to 2009 values for consistency yields a cost of 20.66 USD per treated person per year [32].

#### Baseline SIR model parameter values

The baseline parameter values used in the SIR model are now described; the following section discusses how the interventions and modelling assumptions affect those values. This information is summarized in Table 2.

- $a_u$ is the number of bites per mosquito per untreated human per day, which is estimated to be 0.25 [33].
- $b_u$ is the transmission probability from infected mosquito to susceptible, untreated human, which is estimated to be 0.022 [33].
- *c* is the transmission probability from infected human to susceptible mosquito, which is estimated to be 0.36 [33].
- $\delta$ is the daily birth rate and death rate. In Kenya in 2014, the estimated annual birth rate was 0.02827 births per person, and the estimated annual death rate was 0.007 deaths per person [34]. Because the model assumes a constant population, the average of these, or 0.017635 births (deaths) per person per year, is converted using compounding to a daily birth (death) rate of $\delta = 4.7895 \times 10^{-5}.$
- $\gamma _u$ is the recovery rate for untreated people. Filipe et al. estimate the average infectious period for untreated people to be 180 days, making $\gamma _u = \frac{1}{180}$ [35].
- $m_u$ is the mosquito density (number of mosquitoes per untreated human), which is estimated to be 20 [13].
- $\mu$ is the mosquito mortality rate, estimated to be 0.095 days$^{-1}$ [35, 36].
- $\omega$ is the duration of immunity without reinfection. The value $\omega = 274$ days, is based on an estimate that immunity lasts between 6 and 12 months [37].
- *q* is the treatment coverage, the percentage of the population that receives a treatment and uses it correctly. Three levels of treatment coverage for each intervention are considered: high (60 %), medium (40 %), and low (20 %).
- $\tau$ is the incubation period in the mosquito, estimated to be 10 days [36].
- *x* is the fraction of the population that is age 4 years or younger, which was approximately 14.6 % in Kenya in 2014 [34].

The expressions given in Eqs. (16), (17), (18) and (19) are used to determine the force of infection ($h_u$ and $h_t$) and the recovery rate ($\rho _u$ and $\rho _t$).

**Table 2:** Malaria transmission parameter values for the baseline, untreated case (corresponding to the subscript “*u*”) and treatment cases (corresponding to the subscript “*t*”)

Symbol	Description	Baseline value (untreated)	Treatment value				
			LLIN	IRS	IPT	ACT	Vaccine
$a_u$, $a_t$	Bites per mosquito per human per day	0.25 [33]	$a_u(1-\beta )$ [15]				
$b_u$, $b_t$	Transmission efficacy from infected mosquito to susceptible, untreated human	0.022 [33]			0.0047		0.005
$\beta$	Proportion of bites that would occur while sleeping		0.8 [15]				
*c*	Transmission efficacy from infected human to mosquito	0.36 [33]					
$\delta$	Daily birth rate and death rate assuming constant population	$4.7895*10^{-5}$ [34]					
$\gamma _u$, $\gamma _t$	Recovery rate in humans	$\frac{1}{180}$ [35]				$\frac{1}{10}$	$\frac{1}{5.5}$
$m_u$, $m_t$	Mosquitoes per human	20 [13]	$m_u(1-\beta \chi _{LLIN})$	$m_{u,IRS}=m_u(1-q\chi _{IRS_u})$ $m_{t}=m_u(1-\chi _{IRS_t})$ [38]			
$\mu$	Mosquito mortality rate	0.095 [35, 36]					
$\omega$	Duration of immunity without reinfection	274 days [37]					
*q*	Treatment coverage	0.2, 0.4 or 0.6					
$\tau$	Incubation period in mosquito	10 days [36]					
*x*	Fraction of the population	0.146 [34]					
	0–4 years of age						
$\chi _{LLIN}$	Probability of mosquito mortality when exposed to a treated net		0.8 [14]				
$\chi _{IRS_t}$	Percent reduction in mosquito density in a house treated with IRS			0.95 [38]			
$\chi _{IRS_u}$	Percent reduction in mosquito density in an untreated house when all nearby houses are treated with IRS			0.5 [38]			

In the baseline value column, an empty space means that the parameter does not apply to the baseline, untreated case. In the treatment value columns, an empty space means that the parameter is unchanged by that particular intervention

Note that the malaria transmission parameters, $a_u$, $b_u$, *c*, and $m_u$, are very location-specific (see [36], p. 409). Adapting this model to any location would require re-estimating these parameters.

#### Effects of interventions on SIR parameters

Each intervention, or combination of interventions, is modelled as affecting a subset of the above parameters.

LLINs protect individual users by decreasing the biting rate, $a_t$, and by killing mosquitoes that contact the insecticidal nets, thus decreasing $m_t$. The values of $a_t$ and $m_t$ are estimated as follows:

- Let $\beta$ be the proportion of mosquito exposure that occurs during sleeping hours.
- Let $\chi _{LLIN}$ be the probability of mortality for a mosquito exposed to a treated net.
- As before, let $m_u$ be the baseline mosquito density absent any treatment.

Then Killeen et al. [15] derive the value of $a_t$ for people using LLIN as

23 $$\begin{aligned} a_t = a_u(1-\beta ), \end{aligned}$$

and the value of $m_t$ for people using LLIN as

24 $$\begin{aligned} m_t = m_u ( 1 - \beta \chi _{LLIN}). \end{aligned}$$

Although Fig. 5 in reference [15] shows a slight increase in overall protection for the treated class as a function of *q*, this increase is modest in the range of *q* considered here, and so $m_t$ is assumed to be independent of *q*. Additionally, Killeen et al. suggest that as treatment coverage increases in a population, even non-users of LLINs benefit from decreased mosquito density. However, the authors were unable to find empirical data to support a robust model of mosquito density in the untreated population as a function of treatment coverage; therefore, the model assumes that the untreated population experiences the baseline mosquito density, $m_u$, for all values of *q*.

To determine the new biting rate, $a_t$, and the new mosquito density, $m_t$, for the treated classes, Eqs. (23) and (24), respectively, are used with $\beta = 0.8$ [15] and $\chi _{LLIN} = 0.8$ [14], and with $a_u = 0.25$, and $m_u =20$ as given earlier.

IRS decreases the number of mosquitoes per treated human, $m_t$, in a similar manner as LLINs. Moreover, IRS can also decrease the mosquito density in untreated areas close to treated areas; thus, $m_u$ is also affected by IRS [38]. Let $\chi _{IRS_t}$ be the reduction in mosquito density in a house treated with IRS, and let $\chi _{IRS_u}$ be the reduction in mosquito density in an untreated house when the treatment coverage is 100 % in a nearby area. Then the value of $m_t$ for a house treated with IRS is

25 $$\begin{aligned} m_t = m_u ( 1 - \chi _{IRS_t}) \end{aligned}$$

The value of $m_u$ when IRS is used at coverage *q* is estimated (based on the empirical results of Zhou et al. [38]) to be

26 $$\begin{aligned} m_{u_{IRS}} = m_u ( 1 - q \chi _{IRS_u}). \end{aligned}$$

As [38] report that the mosquito density in treated areas decreases by 95 %, and the mosquito density in untreated areas decreases by 50 % when the coverage in nearby treated areas is 100 %, the simulations use $\chi _{IRS_t} = 0.95$ and $\chi _{IRS_u} = 0.5$, $m_u = 20$ as given earlier, and *q* equal to the coverage associated with the selected action.

IPT decreases the probability, $b_t$, that a susceptible person becomes infected after a bite from an infectious mosquito. The authors were unable to find an estimate in the literature for the amount by which the transmission efficacy, $b_t$, decreases when a person is using IPT. However, data from several studies reported by Aponte et al. indicate that the protective efficacy against malaria in infants of 1 year of IPT is roughly 30 % [6]. As this should roughly correspond to the percentage decrease in new malaria infections observed in the SIR model output, the model was calibrated by solving the system of differential equations for a range of values for $b_t$ and selecting the value of $b_t$ that achieves a 30 % reduction in new malaria infections. The value $b_t =0.0047$ achieves this percentage reduction.

ACT dramatically reduces the length of time a malaria patient is carrying infectious gametocytes in her blood, possibly down to a mean infectious period of 10 days, so the simulations use $\gamma _t = \frac{1}{10}$ days$^{-1}$ [16, 17, 28].

Vaccine, like IPT, decreases the probability, $b_t$, that a susceptible person becomes infected after a bite from an infectious mosquito. Additionally, a vaccine could increase the recovery rate, $\gamma _t$, by exposing the immune system to parasite proteins or decreasing the amount of parasites that reach the blood stage initially [10]. Olotu et al. report clinical trial results suggesting that the 4-year reduction in malaria episodes among vaccinated children is 23.5–24.3 % [39]. As this should roughly correspond to the percentage decrease in new malaria infections observed in the SIR model output, the model was calibrated by solving the system of differential equations for a range of values for both $b_t$ and $\gamma _t$ and selecting the combination that achieves a roughly 24 % reduction in new malaria infections. Choosing $b_t = 0.005$ and $\gamma _t = \frac{1}{5.5}$ days$^{-1}$ achieves this percentage reduction.

Possible actions that can be selected by the optimization model are to deploy no intervention, a single intervention, or a combination of two interventions. Although it is possible to consider combining any pair of interventions, for modelling simplicity, the only pairs of interventions considered are those whose coverage applies to the same segments of the population. Therefore, because IPT and vaccines are assumed in the model to be distributed only to newborns and children under the age of four, while LLIN, ACT and IRS are applied to the general population, the combinations considered are IPT with vaccine, LLIN with ACT, LLIN with IRS, or ACT with IRS.

When two interventions are used in combination, it is assumed that the covered segment of the population receives both treatments, and the uncovered segment receives neither. If the two interventions affect non-overlapping parameter sets, it is assumed the combination intervention will affect the union of both sets of parameters in the same manner as the individual interventions. However, some pairs of interventions act upon the same parameter. For example, in the case of LLIN combined with IRS, the mosquito density, $m_t$ is affected by both interventions. Because it would be too optimistic to assume that the effects of LLIN and IRS are additive, the model makes a more conservative assumption: the smallest values of $a_t$, $m_u$ and $m_t$ offered by either LLIN or IRS are used. For the IPT with vaccine combination, $b_t$, the transmission efficacy from infected mosquito to susceptible, treated human, is reduced by both interventions via different mechanisms, and $\gamma _t$ is increased by the vaccine. Therefore, the smaller of the two $b_t$ values under IPT and vaccine (that given by IPT, of $b_t=0.0047$) and the value of $\gamma _t = \frac{1}{5.5}$ yielded by the vaccine are used.

#### Sensitivity analysis simulations

The optimal sequence of 5-year interventions in a fictitious nation were simulated and analysed. This nation consists of 4500 districts, each having a population of 10,000 (for a total population of 45 million, comparable to that of Kenya in 2014 [34]). An annual budget of $B_t = 33.75$ million USD is used, which corresponds to 0.75 USD per person, per year. (This is comparable to the budget for the President’s Malaria Initiative (PMI) in Kenya, which in 2013 was 34,256,770 USD [40]).

Each of the 4500 districts is characterized as belonging to one of nine geographic regions, which in turn are characterized by one of three distribution regions and one of three climate regions. 500 districts belong to each of the nine possible combinations. Distribution regions categorize districts by how inexpensively (relative to the baseline costs given earlier) interventions can be distributed to the district. Rural and remote areas are likely to experience higher-than-baseline distribution costs due to having worse road infrastructure and lower access to health centres. Centrally located urban areas are likely to experience lower-than-baseline distribution costs. Districts categorized as having low distribution costs are assumed to have intervention costs that are 20 % lower than the baseline values given in Table 1. Districts categorized as having medium distribution costs will incur the baseline intervention costs, and districts categorized as having high distribution costs will incur intervention costs that are 20 % more than the baseline costs given in Table 1.

The three climate regions, dry, moderate and wet, reflect the effect climatic characteristics such as temperature and precipitation can have on mosquito population, and hence on malaria transmission dynamics. The moderate climate scenario assumes the baseline mosquito density given above of $m_u = 20$ mosquitoes per human The dry scenario assumes a mosquito density of $m_u = 5$ mosquitoes per human, and the wet scenario assumes a mosquito density of $m_u=35$ mosquitoes per human. Each region is also characterized by its own initial population distribution amongst the *S*, *I* and *R* classes, which is chosen to be the steady-state population distribution observed when the SIR model is run for a long period of time from a variety of starting population distributions and assuming no intervention. For the moderate climate scenario, the steady-state distribution used for the initial distribution is 15 % susceptible, 15 % infected, and 70 % recovered. The dry scenario uses an initial steady-state distribution of 60 % susceptible, 15 % infected, and 25 % recovered. The wet scenario uses an initial steady-state population distribution of 10 % susceptible, 15 % infected, and 75 % recovered. (For computational tractability, a population state resolution of five percentiles is used, and the SIR population state is rounded to the nearest 5 %, while requiring that the percentages over all compartments sum to one).

Three efficacy scenarios are also considered to test the sensitivity of the model to the inherent uncertainty in the efficacy of the interventions. The baseline efficacy scenario uses the baseline parameter estimates described earlier and shown in Table 2. The pessimistic efficacy scenario assumes each parameter value for the treated class is 30 % “worse” than its baseline value, where “worse” means leading to greater malaria infections. The optimistic efficacy scenario assumes each parameter value for the treated class is 30 % “better” than its baseline value. In the case where making a treatment parameter 30 % worse than its baseline value ends up making it worse than the untreated baseline value, the value is capped at the untreated baseline; in this way, a situation is avoided in which the treated class might artificially experience more malaria cases than the untreated class.

## Results and discussion

The results of running the model on this data are now presented. Because a simplified SIR model is used to estimate the social costs of malaria as a function of interventions distributed, the purpose of this model is not to give exact estimates of reductions in person-days of malaria infection, but relative results that can be used by the optimization model to make choices between the various interventions. The model provides qualitative insights about trends in the optimal interventions as certain parameters vary; these qualitative insights can then be used to offer high-level policy recommendations, as described below.

**Fig. 1:**
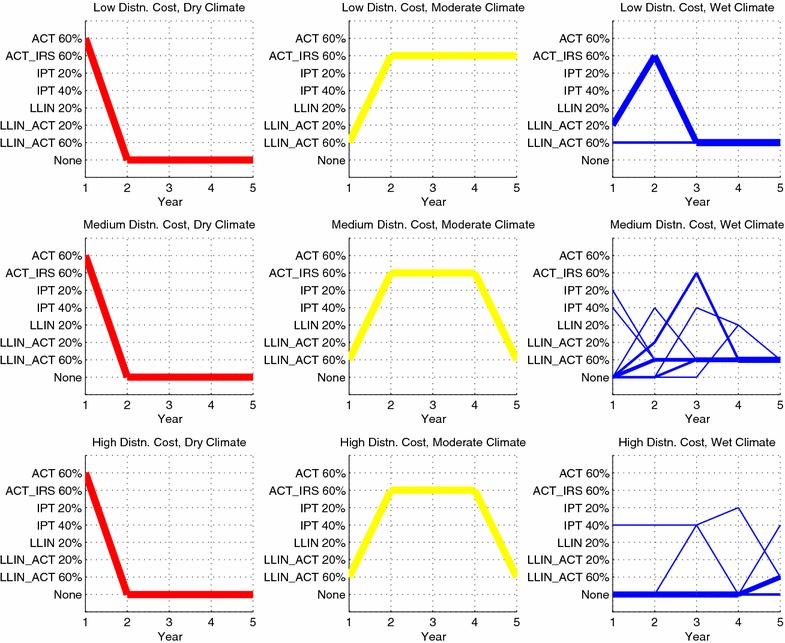
Sequence of interventions over a 5-year time horizon in the baseline efficacy scenario. The thickness of each line is proportional to the number of districts assigned a given sequence. Intervention sequences assigned to only a single district have been omitted from the figure (five districts omitted in total). The total person-days of malaria infection in this scenario is 4.506 billion

Table 3 and Fig. 1 provide the sequence of interventions allocated in each of the nine geographic regions (low, medium or high distribution costs, crossed with dry, moderate or wet climate) over a 5-year horizon in the baseline efficacy case. The total person-days of malaria infection is found to be 4.506 billion. In Fig. 1, the first row of figures corresponds to low distribution costs, the second row to medium distribution costs and the third row to high distribution costs. Likewise, each column of figures corresponds to the same climate region (dry in red, moderate in yellow, wet in blue, from left to right). The vertical axis on each graph lists in alphabetical order the interventions selected by the model at least once in at least one geographic region and the associated coverage. The thickness of a path is proportional to the number of districts (out of 500) that were assigned that sequence of interventions. Table 3 gives the number of districts of each type that are assigned a particular sequence of interventions, as well as the resulting (*S*, *I*, *R*) population state after each year.

**Table 3:** Optimal 5-year (Y1 through Y5) sequences of interventions for the baseline efficacy scenario

Geographic region	Number of districts	Initial population state	Y1 intervention (end pop. state)	Y2 intervention (end pop. state)	Y3 intervention (end pop. state)	Y4 intervention (end pop. state)	Y5 intervention (end pop. state)
(D, L)	500	(60, 15, 25)	ACT 60 %	None	None	None	None
			(90, 0, 10)	(95, 0, 5)	(100, 0, 0)	(100, 0, 0)	(100, 0, 0)
(D, M)	500	(60, 15, 25)	ACT 60 %	None	None	None	None
			(90, 0, 10)	(95, 0, 5)	(100, 0, 0)	(100, 0, 0)	(100, 0, 0)
(D, H)	500	(60, 15, 25)	ACT 60 %	None	None	None	None
			(90, 0, 10)	(95, 0, 5)	(100, 0, 0)	(100, 0, 0)	(100, 0, 0)
(M, L)	500	(15, 15, 70)	LLIN_ACT 60 %	ACT_IRS 60 %	ACT_IRS 60 %	ACT_IRS 60 %	ACT_IRS 60 %
			(65, 5, 30)	(80, 5, 15)	(85, 5, 10)	(85, 5, 10)	(85, 5, 10)
(M, M)	500	(15, 15, 70)	LLIN_ACT 60 %	ACT_IRS 60 %	ACT_IRS 60 %	ACT_IRS 60 %	LLIN_ACT 60 %
			(65, 5, 30)	(80, 5, 15)	(85, 5, 10)	(85, 5, 10)	(75, 10, 15)
(M, H)	500	(15, 15, 70)	LLIN_ACT 60 %	ACT_IRS 60 %	ACT_IRS 60 %	ACT_IRS 60 %	LLIN_ACT 60 %
			(65, 5, 30)	(80, 5, 15)	(85, 5, 10)	(85, 5, 10)	(75, 10, 15)
(W, L)	395	(10, 15, 75)	LLIN_ACT 20 %	ACT_IRS 60 %	LLIN_ACT 60 %	LLIN_ACT 60 %	LLIN_ACT 60 %
			(25, 10, 65)	(65, 5, 30)	(60, 10, 30)	(60, 10, 30)	(60, 10, 30)
(W, L)	105	(10, 15, 75)	LLIN_ACT 60 %	LLIN_ACT 60 %	LLIN_ACT 60 %	LLIN_ACT 60 %	LLIN_ACT 60 %
			(60, 5, 35)	(60, 10, 30)	(60, 10, 30)	(60, 10, 30)	(60, 10, 30)
(W, M)	276	(10, 15, 75)	None	LLIN_ACT 60 %	LLIN_ACT 60 %	LLIN_ACT 60 %	LLIN_ACT 60 %
			(10, 15, 75)	(60, 5, 35)	(60, 10, 30)	(60, 10, 30)	(60, 10, 30)
(W, M)	136	(10, 15, 75)	None	LLIN_ACT 20 %	ACT_IRS 60 %	LLIN_ACT 60 %	LLIN_ACT 60 %
			(10, 15, 75)	(25, 10, 65)	(65, 5, 30)	(60, 10, 30)	(60, 10, 30)
(W, M)	59	(10, 15, 75)	None	None	LLIN_ACT 60 %	LLIN_ACT 60 %	LLIN_ACT 60 %
			(10, 15, 75)	(10, 15, 75)	(60, 5, 35)	(60, 10, 30)	(60, 10, 30)
(W, M)	22	(10, 15, 75)	None	None	None	LLIN 20 %	LLIN_ACT 60 %
			(10, 15, 75)	(10, 15, 75)	(10, 15, 75)	(25, 10, 65)	(55, 10, 35)
(W, M)	3	(10, 15, 75)	None	None	IPT 40 %	LLIN 20 %	LLIN_ACT 60 %
			(10, 15, 75)	(10, 15, 75)	(10, 15, 75)	(25, 10, 65)	(55, 10, 35)
(W, M)	2	(10, 15, 75)	None	IPT 40 %	LLIN_ACT 60 %	LLIN_ACT 60 %	LLIN_ACT 60 %
			(10, 15, 75)	(10, 15, 75)	(60, 5, 35)	(60, 10, 30)	(60, 10, 30)
(W, M)	1	(10, 15, 75)	IPT 20 %	LLIN_ACT 60 %	LLIN_ACT 60 %	LLIN_ACT 60 %	LLIN_ACT 60 %
			(10, 15, 75)	(60, 5, 35)	(60, 10, 30)	(60, 10, 30)	(60, 10, 30)
(W, M)	1	(10, 15, 75)	IPT 40 %	LLIN_ACT 60 %	LLIN_ACT 60 %	LLIN_ACT 60 %	LLIN_ACT 60 %
			(10, 15, 75)	(60, 5, 35)	(60, 10, 30)	(60, 10, 30)	(60, 10, 30)
(W, H)	371	(10, 15, 75)	None	None	None	None	LLIN_ACT 60 %
			(10, 15, 75)	(10, 15, 75)	(10, 15, 75)	(10, 15, 75)	(60, 5, 35)
(W, H)	126	(10, 15, 75)	None	None	None	None	None
			(10, 15, 75)	(10, 15, 75)	(10, 15, 75)	(10, 15, 75)	(10, 15, 75)
(W, H)	1	(10, 15, 75)	None	None	None	None	IPT 40 %
			(10, 15, 75)	(10, 15, 75)	(10, 15, 75)	(10, 15, 75)	(10, 15, 75)
(W, H)	1	(10, 15, 75)	None	None	IPT 40 %	None	LLIN_ACT 60 %
			(10, 15, 75)	(10, 15, 75)	(10, 15, 75)	(10, 15, 75)	(60, 5, 35)
(W, H)	1	(10, 15, 75)	IPT 40 %	IPT 40 %	IPT 40 %	IPT 20 %	LLIN_ACT 60 %
			(10, 15, 75)	(10, 15, 75)	(10, 15, 75)	(10, 15, 75)	(60, 5, 35)

Geographic region refers to the climate region and distribution cost pair, where the climate region is dry (D), moderate (M) or wet (W), and the distribution cost is low (L), medium (M) or high (H). Number of districts refers to the number of districts that were assigned a given trajectory. Initial population state is the starting (*S*, *I*, *R*) percentages of the region; the end population state for a given year is the resulting (*S*, *I*, *R*) state after distributing the corresponding intervention. The total person-days of malaria infection in this scenario is 4.506 billion

Note that for dry climate regions, the sequence of interventions is the same regardless of distribution cost, namely, ACT is distributed to 60 % of the population in year 1, and then no subsequent interventions are distributed in years 2–5. The reason for this is that distributing ACT at a coverage of 60 % in year 1 eradicates (at least subject to rounding at a resolution of 5 %) malaria, driving the infected proportion of the population to zero. Per Eqs. (16) and (17), the force of infection is zero when the infected population is zero, and the disease cannot persist.

For the moderate climate regions, all districts are assigned ACT combined with LLIN at 60 % coverage in the first year, followed by ACT in combination with either LLIN or IRS at 60 % coverage in subsequent years.

The sequence of interventions assigned in the wet regions at first glance appears more interesting. First, note that only low distribution cost districts, along with only two medium distribution cost and one high distribution cost districts, receive any intervention during the first year. 126 high distribution cost districts never receive any intervention in any of the 5 years. This is likely due to the budget constraint forcing the model to prioritize eliminating malaria in the dry climate regions during year 1 and leaving the harder-to-access wet regions largely untreated. In those wet districts receiving interventions, the chosen interventions are primarily LLIN with ACT at 60 % coverage, but as Table 3 shows, these interventions do little to reduce the prevalence of malaria in the population. An apparent steady-state consists of 10 % of the population in the infected state even after several years of 60 % coverage of LLIN with ACT. Thus, it can be inferred that combating malaria in the wet regions is not possible with the interventions considered at coverage percentages up to 60 %. As will be shown later, increasing the maximum coverage to 80 % is necessary for reducing malaria in wet regions.

### Effect of treatment efficacy

**Fig. 2:**
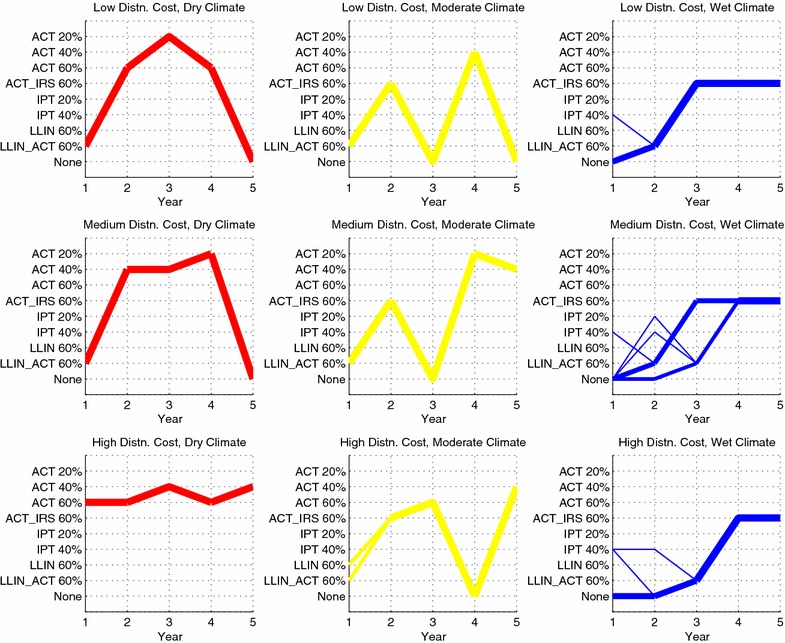
Sequence of interventions over a 5-year time horizon in the optimistic efficacy scenario. The thickness of each line is proportional to the number of districts assigned a given sequence. Intervention sequences assigned to only a single district have been omitted from the figure (four districts omitted in total). The total person-days of malaria infection in this scenario is 2.977 billion

**Fig. 3:**
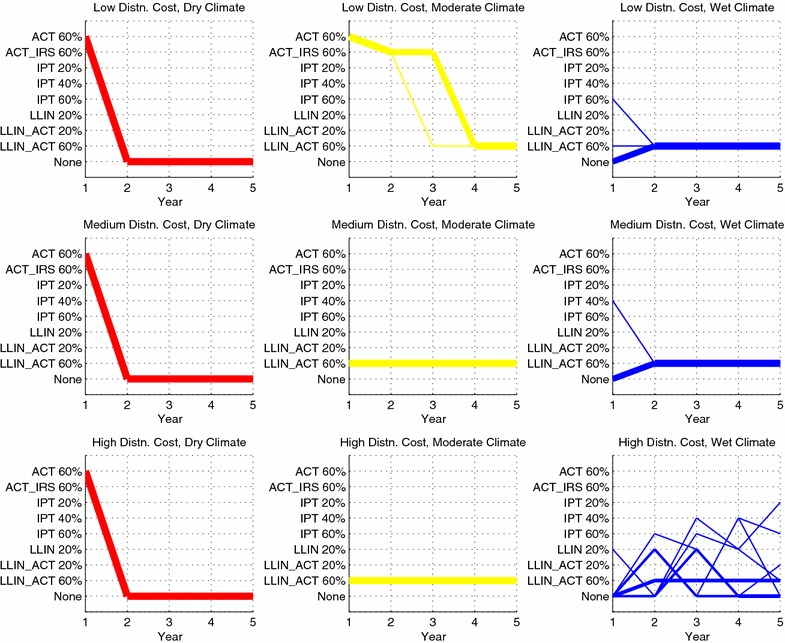
Sequence of interventions over a 5-year time horizon in the pessimistic efficacy scenario. The thickness of each line is proportional to the number of districts assigned a given sequence. Intervention sequences assigned to only a single district have been omitted from the figure (five districts omitted in total). The total person-days of malaria infection in this scenario is 5.080 billion

The sequence of interventions allocated in the nine geographic regions under the optimistic and pessimistic efficacy scenarios, in which the disease transmission parameter values for the treated class are adjusted up or down by 30 %, can also be examined. The optimistic case is depicted in Fig. 2, with the full set of results given in Table 4, and the results for the pessimistic case are given in Fig. 3 and Table 5.

**Table 4:** Optimal 5-year (Y1 through Y5) sequences of interventions for the optimistic efficacy scenario

Geographic region	Number of districts	Initial population state	Y1 intervention (end pop. state)	Y2 intervention (end pop. state)	Y3 intervention (end pop. state)	Y4 intervention (end pop. state)	Y5 intervention (end pop. state)
(D, L)	500	(60, 15, 25)	LLIN_ACT 60 %	ACT 60 %	ACT 20 %	ACT 60 %	None
			(90, 0, 10)	(95, 0, 5)	(100, 0, 0)	(100, 0, 0)	(100, 0, 0)
(D, M)	500	(60, 15, 25)	LLIN_ACT 60 %	ACT 40 %	ACT 40 %	ACT 20 %	None
			(90, 0, 10)	(95, 0, 5)	(100, 0, 0)	(100, 0, 0)	(100, 0, 0)
(D, H)	500	(60, 15, 25)	ACT 60 %	ACT 60 %	ACT 40 %	ACT 60 %	ACT 40 %
			(85, 5, 10)	(95, 0, 5)	(100, 0, 0)	(100, 0, 0)	(100, 0, 0)
(M, L)	500	(15, 15, 70)	LLIN_ACT 60 %	ACT_IRS 60 %	None	ACT 40 %	None
			(65, 5, 30)	(90, 0, 10)	(95, 0, 5)	(100, 0, 0)	(100, 0, 0)
(M, M)	500	(15, 15, 70)	LLIN_ACT 60 %	ACT_IRS 60 %	None	ACT 20 %	ACT 40 %
			(65, 5, 30)	(90, 0, 10)	(95, 0, 5)	(100, 0, 0)	(100, 0, 0)
(M, H)	266	(15, 15, 70)	LLIN_ACT 60 %	ACT_IRS 60 %	ACT 60 %	None	ACT 40 %
			(65, 5, 30)	(90, 0, 10)	(95, 0, 5)	(100, 0, 0)	(100, 0, 0)
(M, H)	234	(15, 15, 70)	LLIN 60 %	ACT_IRS 60 %	ACT 60 %	None	ACT 40 %
			(60, 5, 35)	(90, 0, 10)	(95, 0, 5)	(100, 0, 0)	(100, 0, 0)
(W, L)	497	(10, 15, 75)	None	LLIN_ACT 60 %	ACT_IRS 60 %	ACT_IRS 60 %	ACT_IRS 60 %
			(10, 15, 75)	(60, 5, 35)	(80, 5, 15)	(75, 10, 15)	(75, 10, 15)
(W, L)	3	(10, 15, 75)	IPT 40 %	LLIN_ACT 60 %	ACT_IRS 60 %	ACT_IRS 60 %	ACT_IRS 60 %
			(10, 15, 75)	(60, 5, 35)	(80, 5, 15)	(75, 10, 15)	(75, 10, 15)
(W, M)	324	(10, 15, 75)	None	LLIN_ACT 60 %	ACT_IRS 60 %	ACT_IRS 60 %	ACT_IRS 60 %
			(10, 15, 75)	(60, 5, 35)	(80, 5, 15)	(75, 10, 15)	(75, 10, 15)
(W, M)	172	(10, 15, 75)	None	None	LLIN_ACT 60 %	ACT_IRS 60 %	ACT_IRS 60 %
			(10, 15, 75)	(10, 15, 75)	(60, 5, 35)	(80, 5, 15)	(75, 10, 15)
(W, M)	2	(10, 15, 75)	IPT 40 %	LLIN_ACT 60 %	ACT_IRS 60 %	ACT_IRS 60 %	ACT_IRS 60 %
			(10, 15, 75)	(60, 5, 35)	(80, 5, 15)	(75, 10, 15)	(75, 10, 15)
(W, M)	1	(10, 15, 75)	None	IPT 20 %	LLIN_ACT 60 %	ACT_IRS 60 %	ACT_IRS 60 %
			(10, 15, 75)	(10, 15, 75)	(60, 5, 35)	(80, 5, 15)	(75, 10, 15)
(W, M)	1	(10, 15, 75)	None	IPT 40 %	LLIN_ACT 60 %	ACT_IRS 60 %	ACT_IRS 60 %
			(10, 15, 75)	(10, 15, 75)	(60, 5, 35)	(80, 5, 15)	(75, 10, 15)
(W, H)	498	(10, 15, 75)	None	None	LLIN_ACT 60 %	ACT_IRS 60 %	ACT_IRS 60 %
			(10, 15, 75)	(10, 15, 75)	(60, 5, 35)	(80, 5, 15)	(75, 10, 15)
(W, H)	1	(10, 15, 75)	IPT 40 %	None	LLIN_ACT 60 %	ACT_IRS 60 %	ACT_IRS 60 %
			(10, 15, 75)	(10, 15, 75)	(60, 5, 35)	(80, 5, 15)	(75, 10, 15)
(W, H)	1	(10, 15, 75)	IPT 40 %	IPT 40 %	LLIN_ACT 60 %	ACT_IRS 60 %	ACT_IRS 60 %
			(10, 15, 75)	(10, 15, 75)	(60, 5, 35)	(80, 5, 15)	(75, 10, 15)

Geographic region refers to the climate region and distribution cost pair, where the climate region is dry (D), moderate (M) or wet (W), and the distribution cost is low (L), medium (M) or high (H). Number of districts refers to the number of districts that were assigned a given trajectory. Initial population state is the starting (*S*, *I*, *R*) percentages of the region; the end population state for a given year is the resulting (*S*, *I*, *R*) state after distributing the corresponding intervention. The total person-days of malaria infection in this scenario is 2.977 billion

In the optimistic case, note a reduction in person-days of malaria infection from 4.506 billion to 2.977 billion, or 34 %. In year 1, the model focuses on eradicating (subject to rounding error) malaria in the dry and moderate climate regions by allocating LLIN, ACT or the two in combination. All but seven of the 1,500 wet climate districts receive no intervention in year 1. In subsequent years, the wet climate districts receive 60 % coverage of LLIN with ACT, and ACT with IRS, with a handful of wet climate districts receiving IPT or no intervention during years 1 and 2. Although it appears that the dry and moderate regions are also receiving interventions during years 2 through 5, this is an artifact of the optimization model: once the infectious population is driven to zero, there is no value in distributing further interventions; the model is allocating interventions in these regions simply to use up the available budget. Moreover, the prevalence of malaria in the wet regions appears to stabilize around 10 % in year 5. This is further indication that 60 % coverage is not sufficient to diminish malaria prevalence in the wet regions, even if the interventions are assumed to be highly effective.

**Table 5:** Optimal 5-year (Y1 through Y5) sequences of interventions for the pessimistic efficacy scenario

Geographic region	Number of districts	Initial population state	Y1 intervention (end pop. state)	Y2 intervention (end pop. state)	Y3 intervention (end pop. state)	Y4 intervention (end pop. state)	Y5 intervention (end pop. state)
(D, L)	500	(60, 15, 25)	ACT 60 %	None	None	None	None
			(90, 0, 10)	(95, 0, 5)	(100, 0, 0)	(100, 0, 0)	(100, 0, 0)
(D, M)	500	(60, 15, 25)	ACT 60 %	None	None	None	None
			(90, 0, 10)	(95, 0, 5)	(100, 0, 0)	(100, 0, 0)	(100, 0, 0)
(D, H)	500	(60, 15, 25)	ACT 60 %	None	None	None	None
			(90, 0, 10)	(95, 0, 5)	(100, 0, 0)	(100, 0, 0)	(100, 0, 0)
(M, L)	494	(15, 15, 70)	ACT 60 %	ACT_IRS 60 %	ACT_IRS 60 %	LLIN_ACT 60 %	LLIN_ACT 60 %
			(60, 5, 35)	(80, 5, 15)	(75, 10, 15)	(70, 10, 20)	(70, 10, 20)
(M, L)	6	(15, 15, 70)	ACT 60 %	ACT_IRS 60 %	LLIN_ACT 60 %	LLIN_ACT 60 %	LLIN_ACT 60 %
			(60, 5, 35)	(80, 5, 15)	(75, 10, 15)	(70, 10, 20)	(70, 10, 20)
(M, M)	500	(15, 15, 70)	LLIN_ACT 60 %	LLIN_ACT 60 %	LLIN_ACT 60 %	LLIN_ACT 60 %	LLIN_ACT 60 %
			(65, 5, 30)	(75, 10, 15)	(70, 10, 20)	(70, 10, 20)	(70, 10, 20)
(M, H)	500	(15, 15, 70)	LLIN_ACT 60 %	LLIN_ACT 60 %	LLIN_ACT 60 %	LLIN_ACT 60 %	LLIN_ACT 60 %
			(65, 5, 30)	(75, 10, 15)	(70, 10, 20)	(70, 10, 20)	(70, 10, 20)
(W, L)	464	(10, 15, 75)	None	LLIN_ACT 60 %	LLIN_ACT 60 %	LLIN_ACT 60 %	LLIN_ACT 60 %
			(10, 15, 75)	(60, 5, 35)	(60, 10, 30)	(60, 10, 30)	(60, 10, 30)
(W, L)	35	(10, 15, 75)	LLIN_ACT 60 %	LLIN_ACT 60 %	LLIN_ACT 60 %	LLIN_ACT 60 %	LLIN_ACT 60 %
			(60, 5, 35)	(60, 10, 30)	(60, 10, 30)	(60, 10, 30)	(60, 10, 30)
(W, L)	1	(10, 15, 75)	IPT 60 %	LLIN_ACT 60 %	LLIN_ACT 60 %	LLIN_ACT 60 %	LLIN_ACT 60 %
			(10, 15, 75)	(60, 5, 35)	(60, 10, 30)	(60, 10, 30)	(60, 10, 30)
(W, M)	499	(10, 15, 75)	None	LLIN_ACT 60 %	LLIN_ACT 60 %	LLIN_ACT 60 %	LLIN_ACT 60 %
			(10, 15, 75)	(60, 5, 35)	(60, 10, 30)	(60, 10, 30)	(60, 10, 30)
(W, M)	1	(10, 15, 75)	IPT 40 %	LLIN_ACT 60 %	LLIN_ACT 60 %	LLIN_ACT 60 %	LLIN_ACT 60 %
			(10, 15, 75)	(60, 5, 35)	(60, 10, 30)	(60, 10, 30)	(60, 10, 30)
(W, H)	241	(10, 15, 75)	None	LLIN_ACT 60 %	LLIN_ACT 60 %	LLIN_ACT 60 %	LLIN_ACT 60 %
			(10, 15, 75)	(60, 5, 35)	(60, 10, 30)	(60, 10, 30)	(60, 10, 30)
(W, H)	140	(10, 15, 75)	None	None	LLIN 20 %	None	None
			(10, 15, 75)	(10, 15, 75)	(20, 10, 70)	(5, 15, 80)	(10, 15, 75)
(W, H)	109	(10, 15, 75)	None	LLIN 20 %	None	None	None
			(10, 15, 75)	(20, 10, 70)	(5, 15, 80)	(10, 15, 75)	(10, 15, 75)
(W, H)	3	(10, 15, 75)	LLIN 20 %	None	IPT 40 %	LLIN 20 %	LLIN_ACT 60 %
			(20, 10, 70)	(5, 15, 80)	(10, 15, 75)	(20, 10, 70)	(55, 10, 35)
(W, H)	2	(10, 15, 75)	None	IPT 60 %	LLIN 20 %	None	None
			(10, 15, 75)	(10, 15, 75)	(20, 10, 70)	(5, 15, 80)	(10, 15, 75)
(W, H)	2	(10, 15, 75)	None	LLIN 20 %	None	IPT 40 %	IPT 60 %
			(10, 15, 75)	(20, 10, 70)	(5, 15, 80)	(10, 15, 75)	(10, 15, 75)
(W, H)	1	(10, 15, 75)	None	None	IPT 60 %	LLIN 20 %	IPT 20 %
			(10, 15, 75)	(10, 15, 75)	(10, 15, 75)	(20, 10, 70)	(10, 15, 75)
(W, H)	1	(10, 15, 75)	None	IPT 60 %	LLIN 20 %	None	LLIN_ACT 20 %
			(10, 15, 75)	(10, 15, 75)	(20, 10, 70)	(5, 15, 80)	(25, 10, 65)
(W, H)	1	(10, 15, 75)	None	LLIN 20 %	None	IPT 40 %	None
			(10, 15, 75)	(20, 10, 70)	(5, 15, 80)	(10, 15, 75)	(10, 15, 75)

Geographic region refers to the climate region and distribution cost pair, where the climate region is dry (D), moderate (M) or wet (W), and the distribution cost is low (L), medium (M) or high (H). Number of districts refers to the number of districts that were assigned a given trajectory. Initial population state is the starting (*S*, *I*, *R*) percentages of the region; the end population state for a given year is the resulting (*S*, *I*, *R*) state after distributing the corresponding intervention. The total person-days of malaria infection in this scenario is 5.080 billion

In the pessimistic case, note an increase in person-days of malaria infection from 4.506 billion to 5.080 billion, or 13 %. In year 1, resources are focused on rapidly reducing infections in the dry regions, by allocating 60 % coverage of ACT; remaining resources in year 1 are focused on the moderate climate regions. In subsequent years, the moderate regions receive 60 % coverage of ACT combined with either IRS or LLIN; the remaining budget is used to allocate a variety of interventions in the wet regions. The takeaway message here is that to minimize person-days of malaria infection, the model chooses to focus resources on the dry and moderate climate regions; any remaining budget is allocated to the wet regions.

### Effect of coverage

If a maximum coverage of 80 % (including both distribution and compliance) is attainable, the results change dramatically. Table 6 gives the sequence of interventions assuming a baseline efficacy and possible coverage levels of 40 %, 60 and 80 % (rather than 20, 40 and 60 % used earlier), and using the same annual budget of 33.75 million USD. In all geographic regions, the infected population is driven to zero within 2 years, indicating that malaria is effectively eradicated (at least subject to rounding at a resolution of 5 %). Thus, achieving high coverage is crucial to rapid eradication of malaria, even with the annual budget held constant. Although intervention costs rise in proportion to the treatment coverage, the fixed budget is able to achieve markedly lower person-days of malaria infection when 80 % coverage is achievable as opposed to only 60 % (1.139 billion person days, as opposed to 4.506 billion person days,) although some of this effect is likely due to rounding at resolution 5 %. Thus, giving a smaller number of cities a higher coverage is more effective than giving a larger number of cities a lower coverage. Moreover, it is only when 80 % coverage is attainable that the prevalence of malaria is reduced in the wet climate regions.

**Table 6:** Optimal 5-year (Y1 through Y5) sequences of interventions for the baseline efficacy scenario when the maximum coverage available for each intervention is 80 %

Geographic region	Number of districts	Initial population state	Y1 intervention (end pop. state)	Y2 intervention (end pop. state)	Y3 intervention (end pop. state)	Y4 intervention (end pop. state)	Y5 intervention (end pop. state)
(D, L)	500	(60, 15, 25)	ACT 80 %	ACT 60 %	None	ACT 40 %	ACT 40 %
			(90, 0, 10)	(95, 0, 5)	(100, 0, 0)	(100, 0, 0)	(100, 0, 0)
(D, M)	500	(60, 15, 25)	ACT 80 %	ACT 80 %	ACT 80 %	ACT 40 %	ACT 60 %
			(90, 0, 10)	(95, 0, 5)	(100, 0, 0)	(100, 0, 0)	(100, 0, 0)
(D, H)	500	(60, 15, 25)	ACT 80 %	None	ACT 80 %	ACT 40 %	None
			(90, 0, 10)	(95, 0, 5)	(100, 0, 0)	(100, 0, 0)	(100, 0, 0)
(M, L)	500	(15, 15, 70)	ACT 80 %	None	ACT 80 %	ACT 80 %	None
			(75, 0, 25)	(95, 0, 5)	(100, 0, 0)	(100, 0, 0)	(100, 0, 0)
(M, M)	500	(15, 15, 70)	ACT 80 %	ACT 40 %	ACT 60 %	ACT 80 %	ACT 40 %
			(75, 0, 25)	(95, 0, 5)	(100, 0, 0)	(100, 0, 0)	(100, 0, 0)
(M, H)	404	(15, 15, 70)	IPT 40 %	LLIN_ACT 80 %	ACT 40 %	ACT 80 %	None
			(20, 15, 65)	(80, 0, 20)	(95, 0, 5)	(100, 0, 0)	(100, 0, 0)
(M, H)	92	(15, 15, 70)	ACT 80 %	ACT 60 %	ACT 40 %	None	None
			(75, 0, 25)	(95, 0, 5)	(100, 0, 0)	(100, 0, 0)	(100, 0, 0)
(M, H)	3	(15, 15, 70)	None	LLIN_ACT 80 %	ACT 40 %	ACT 80 %	None
			(15, 20, 65)	(80, 0, 20)	(95, 0, 5)	(100, 0, 0)	(100, 0, 0)
(W, L)	500	(10, 15, 75)	LLIN_ACT 80 %	None	ACT 40 %	ACT 80 %	ACT 80 %
			(75, 0, 25)	(95, 0, 5)	(100, 0, 0)	(100, 0, 0)	(100, 0, 0)
(W, M)	500	(10, 15, 75)	None	ACT_IRS 80 %	None	ACT 60 %	ACT 60 %
			(10, 15, 75)	(80, 0, 20)	(95, 0, 5)	(100, 0, 0)	(100, 0, 0)
(W, H)	354	(10, 15, 75)	None	ACT_IRS 80 %	ACT 40 %	ACT 40 %	None
			(10, 15, 75)	(80, 0, 20)	(95, 0, 5)	(100, 0, 0)	(100, 0, 0)
(W, H)	146	(10, 15, 75)	None	LLIN_ACT 80 %	None	ACT 40 %	None
			(10, 15, 75)	(75, 0, 25)	(95, 0, 5)	(100, 0, 0)	(100, 0, 0)

Geographic region refers to the climate region and distribution cost pair, where the climate region is dry (D), moderate (M) or wet (W), and the distribution cost is low (L), medium (M) or high (H). Number of districts refers to the number of districts that were assigned a given trajectory. Initial population state is the starting (*S*, *I*, *R*) percentages of the region; the end population state for a given year is the resulting (*S*, *I*, *R*) state after distributing the corresponding intervention. The total person-days of malaria infection in this scenario is 1.139 billion, and in all geographic regions, malaria is eradicated over the 5-year time horizon

Because the 80 % coverage eradicates malaria so quickly in the model, the interpretation of the 5-year model results becomes less interesting. For this reason, the remainder of the results will be presented using the original coverage percentages of 20, 40 and 60 %, except where otherwise indicated.

### Effect of budget

**Fig. 4:**
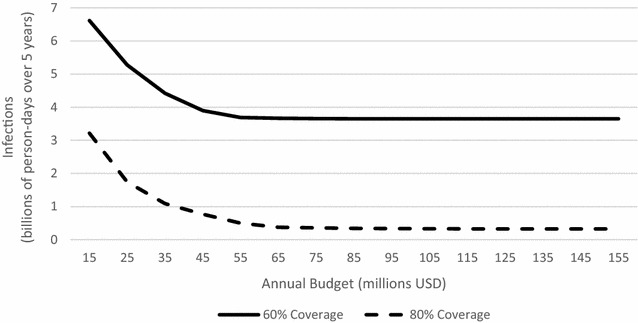
Total person-days of malaria infection over 5-year horizon as a function of annual budget in the baseline efficacy scenario. The *solid line* corresponds to a maximum attainable coverages of 60 %. The *dashed line* corresponds to a maximum attainable coverage of 80 %

Previous work by Dimitrov et al. suggested that increasing the budget would decrease malaria deaths roughly linearly up to a critical budget value, after which there would be diminishing marginal benefit to additional budget expenditures [11]. Using the baseline efficacy scenario, the optimization problem was solved sequentially for budgets ranging from 15 million USD to 155 million USD in increments of 10 million USD, and person-days of malaria infection was plotted against budget. As seen in Fig. 4, the model’s results are consistent with Dimitrov et al. [11]. The person-days of malaria infection decrease linearly with an increase in budget until roughly 55 million USD, after which diminishing marginal returns on budget increases are observed. Moreover, beyond a budget of 85 million USD, there can be no further reduction in person-days of malaria infection. Also shown in Fig. 4 is the plot of person-days of malaria infection in the baseline scenario when coverage up to 80 % is attainable. The graph exhibits the same general shape, but with substantially lower values for the person-days of malaria infection. Moreover, even with a very large budget, 60 % coverage cannot achieve the low prevalence of malaria infection that 80 % coverage can achieve with even a small budget.

**Fig. 5:**
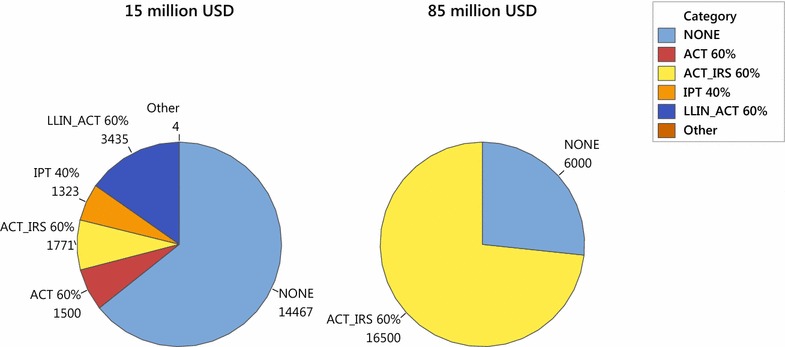
Interventions chosen (in district-years) under a 15 million USD annual budget as compared to an 85 million USD annual budget, aggregated over all geographic regions and a 5-year time horizon in the baseline efficacy scenario

Additionally, the qualitative change in the interventions chosen at different budget levels is evident in Fig. 5. When the budget is 15 million USD, for instance, most districts receive no intervention in most years. Those that do receive 60 % coverage of ACT, either alone or in combination with either IRS or LLIN, or 40 % coverage of IPT. (Although not shown in Fig. 5, the raw results from the simulation reveal that the dry regions receive interventions in year 1, which effectively eradicates malaria in those regions; most of the moderate climate regions receive interventions in most years, with a cluster of high distribution cost districts receiving no intervention in any of the 5 years; and the wet regions typically receive no intervention in most years). When the budget is 85 million USD, Fig. 5 shows that the number of districts receiving no intervention in some year drops sharply. (A closer look at the raw results reveals that all regions receive the maximum of 60 % coverage of ACT in combination with IRS in all years, except for the dry regions, which receive no intervention in years 2 through 5 after malaria has been effectively eradicated).

**Fig. 6:**
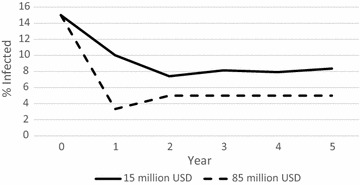
Percentage of the population in the infected class over time under a 15 million USD annual budget (*solid line*) as compared to an 85 million USD annual budget (*dashed line*), aggregated over all geographic regions, in the baseline efficacy scenario

Figure 6 shows the percentage of the total population that is in the infected class at the end of each of the 5 years, when the annual budget is 15 million USD as compared to 85 million USD. A 15 million USD annual budget achieves a drop from 15 % infected to around 8 % infected in steady state. An 85 million USD annual budget achieves an initial drop to around 3 % infected, which levels out to 5 % infected in steady-state. (Note that this contrasts with the results shown previously in Table 6 where if coverage of 80 % is attainable, malaria is eradicated with only a 33.75 million USD budget).

### Role of vaccine

**Fig. 7:**
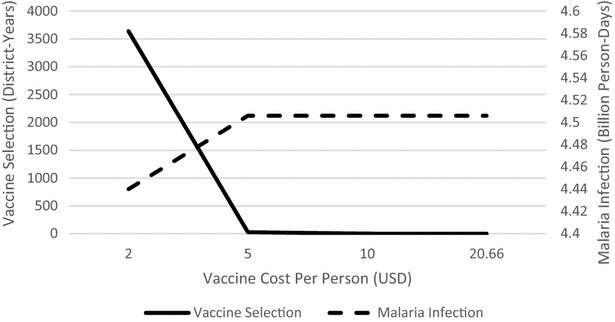
Vaccine selection (in number of district-years) and person-days of malaria infection versus vaccine cost, over 5-year time horizon, in the baseline efficacy scenario

The authors were surprised to observe that in all of the simulations across geographic regions, efficacy scenarios, maximum coverage, and budgets, the vaccine is almost never chosen, either alone or in combination with IPT. Although the annual cost per treated person is quite high (20.66 USD), the fact that it is distributed only to children under the age of four, comprising an estimated 14.6 % of the population, makes its district-wide cost on par with that of the other interventions. The model can be used to understand under what conditions a malaria vaccine would be a cost-effective intervention by systematically lowering its cost and increasing its efficacy. Specifically, the optimization model was solved on all combinations of $b_t = 0.001, 0.005,$ or 0.009, $\gamma _t = \frac{1}{2}, \frac{1}{5.5},$ or $\frac{1}{30}$, and $\text {vaccine cost} = 2, 5, 10,$ or 20.66 USD per person annually. Preliminary analysis of these results showed little influence of either $b_t$ or $\gamma _t$ on the number of times vaccine was selected; for most combinations of $b_t$ and $\gamma _t$, vaccine will be frequently chosen if the cost is 2 or 5 USD and will rarely be chosen if the cost is 10 or 20.66 USD. Therefore, it can be concluded that cost is a driving factor in the choice to select vaccines over other interventions. Figure 7 shows the number of districts receiving vaccine, either alone or in combination with IPT, over the 5-year time horizon as a function of vaccine cost, for the baseline efficacy scenario and using $b_t=0.005,$ and $\gamma _t=\frac{1}{5.5}$. When the vaccine cost is 2 USD per person, it is selected quite often, but is selected far less frequently when the cost is 5 USD. Moreover, it is almost never selected at a cost of 10 or 20.66 USD. Shown in the same figure is the person-days of malaria infection in those same instances. Reducing the vaccine cost from 20.66 USD per person to 2 USD per person yields only a 1.5 % reduction in person-days of malaria infection, from 4.51 billion to 4.44 billion.

### Role of time horizon

**Fig. 8:**
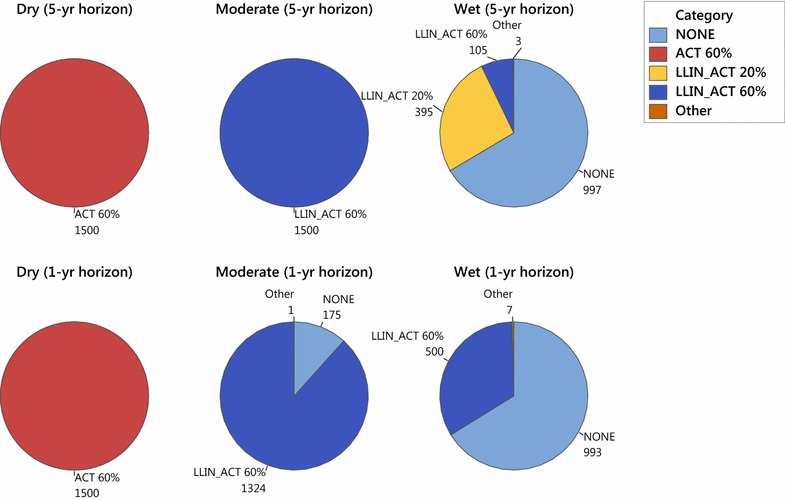
First-year interventions chosen in a 1-year time horizon versus a 5-year time horizon, by climate region, in the baseline efficacy scenario

An important motivation underlying this work is the idea that distributing an intervention changes the malaria transmission dynamics, which could affect future intervention choices. Figure 8 compares the interventions chosen in the first year of the 5-year time horizon to those chosen if the time horizon is only a single year. As mentioned above, in the first year of the 5-year time horizon, resources are focused primarily on the dry and moderate climate regions that receive either ACT alone at 60 % coverage, or ACT in combination with LLIN at 60 % coverage. Most of the medium-to-high distribution cost districts in the wet regions receive no treatment during year 1. Nearly 400 of the low distribution cost districts in the wet regions get 20 % coverage of LLIN with ACT, and the remaining 100 get 60 % coverage of LLIN with ACT. By contrast, if only a 1-year time horizon is used, the solution changes. Dry regions (at all distribution costs) again receive ACT alone at 60 % coverage. Moderate climate regions having low or medium distribution costs receive LLIN in combination with ACT at 60 % coverage, as do 324 moderate climate regions having high distribution costs. However, now 175 moderate climate districts having high distribution costs drop down to no intervention, and in return, all 500 wet climate districts having low distribution costs receive LLIN with ACT at 60 % coverage, which is substantially higher than before. Thus, incorporating a multi-year planning horizon that anticipates changes in the malaria transmission as a result of the interventions distributed does indeed affect the optimal choice of intervention.

### Role of climate

**Fig. 9:**
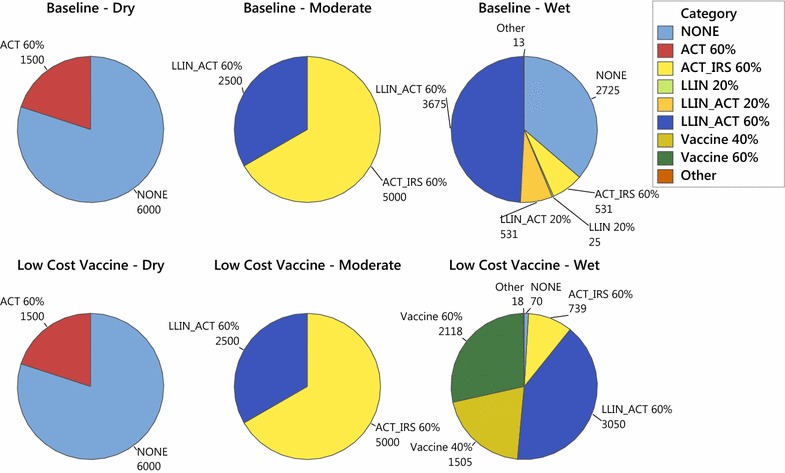
Interventions chosen by climate region when vaccine cost is 20.66 USD per treated person versus a low-cost vaccine having cost 2 USD per treated person, in the baseline efficacy scenario

There is a noticeable qualitative difference in the types of treatments distributed to the three climate regions considered. Aggregating over all years and distribution cost categories for the baseline efficacy scenario, the first row of Fig. 9 shows the number of districts receiving each type of treatment, separated by climate region. Dry regions are adequately served by ACT alone; moderate regions require ACT in combination with either LLIN or IRS; and wet regions get LLIN alone, LLIN with ACT, ACT with IRS, and a handful of others (though as discussed earlier, this variety is likely the result of the model using the available budget because no treatment at 60 % coverage is very successful at reducing malaria in the wet regions).

If a lower-cost vaccine is available at 2 USD per person per year, the second row of Fig. 9 shows that dry regions again receive exclusively no intervention and ACT, and moderate regions again receive exclusively ACT in combination with either IRS or LLIN. Now, however, in the wet regions, the number of years of “no intervention” drops significantly, from 2725 to 70, and vaccine use, at either 40 or 60 % coverage, comprises nearly 50 % of the pie chart.

The takeaway messages are that a single choice of intervention across all climate types is unlikely to be optimal, and that the development of a low-cost malaria vaccine is likely to be of greatest use in regions with high mosquito densities; drier areas are better served by ACT, alone or in combination with IRS and LLIN.

## Conclusions

Because the model is very sensitive to the disease transmission parameter values used, it is best suited for qualitative interpretations about relative benefits of certain interventions. For instance, while common sense might suggest that intervention resources should be focused on wet climate regions with high mosquito counts, the results of the simulations with a maximum coverage of 60 % suggest the opposite: to reduce person-days of malaria infection, it might be better to invest resources on areas where interventions can dramatically reduce the prevalence of malaria, rather than expend resources on areas where malaria is likely to persist, despite best efforts. But if coverage closer to 80 % is attainable, then malaria can be combatted in wet climate regions. Likewise, the model can illustrate the sensitivity of the optimal policy to certain parameters. For example, the optimal sequence of interventions varies by climate type (as represented by mosquito density), suggesting that a one-size-fits-all approach to malaria eradication is not optimal. The sensitivity of the model to parameter assumptions also signals that prior to using the model to guide policy in any given region, the choice of parameter values would first need to be calibrated to match known malaria prevalence rates in the region.

Future refinements of the model could address acquired resistance to interventions, age-dependent immunity, spatial effects of human or mosquito migration, and computational tractability. Drug resistance in malaria parasites and insecticide resistance in mosquitoes are major challenges to control and eradication efforts [41–43]. Implementing resistance in this model would require tracking decreased effectiveness of treatment after use in multiple consecutive years. This would increase the computational challenge of solving the model, however, as the costs and benefits of choosing a particular intervention in a given year would depend not only on the (*S*, *I*, *R*) population state but also on the sequence of interventions chosen in prior years. Likewise, incorporating age-dependent immunity or spatial effects would also require an increase in the number of population compartments in the (*S*, *I*, *R*) model. Already, computational power was limited, even using a 5 % population resolution. The bottleneck appears to be in the formulation of the ILP using AMPL. Although the data file is only 2 MB at 5 % resolution and 11 MB at 2 % resolution, loading the 5 % resolution data file into AMPL took approximately 15 min on a 32-core, 128 GB RAM parallel compute server located in the Harvey Mudd College Mathematics Department, and attempting to load the data file for the 2 % resolution case exceeded the available 128 GB of RAM on the server. Once loaded, the 5 % resolution model was subsequently solved by the CPLEX solver within seconds. The technical staff at the NEOS server (an online server for optimization solvers on which earlier tests were run [44–46]) who are familiar with these modelling languages suggested using a more efficient modelling language than AMPL for formulating the optimization model; this is left as future work.

Given the growing interest in malaria eradication, the WHO Global Malaria Programme cites the need for operations research studies to determine the best intervention strategies in areas where transmission dynamics are changing as malaria is being eliminated. They also present a list of priority research questions that includes questions about safety, access, and community involvement [2]. This paper presents a flexible modelling framework that can guide such decisions. The model permits a multi-year planning horizon over areas characterized by disparate infrastructure and climate. Given inputs of the per-person cost of each intervention and the effects each intervention has on malaria disease transmission parameters, the model provides a sequence of interventions over a fixed time horizon that minimizes person-days of malaria infection subject to an annual budget. Moreover, this model can be adapted to the treatment of other infectious diseases.
